# Chitin Translocation Is Functionally Coupled with Synthesis in Chitin Synthase

**DOI:** 10.3390/ijms252111667

**Published:** 2024-10-30

**Authors:** Suhao Niu, Lei Qi, Xiaoyue Zhang, Dongfang He, Pengwei Li, Hao Wang, Yunchen Bi

**Affiliations:** 1 CAS and Shandong Province Key Laboratory of Experimental Marine Biology, Center for Ocean Mega-Science, Institute of Oceanology, Chinese Academy of Sciences, Qingdao 266071, China; 2 Laboratory for Marine Biology and Biotechnology, Qingdao Marine Science and Technology Center, Qingdao 266237, China; 3 Biomedical Research Center for Structural Analysis, Shandong University, Jinan 250012, China; 4 University of Chinese Academy of Sciences, Beijing 100049, China

**Keywords:** chitin synthase, membrane translocation, processive, glycosyltransferase

## Abstract

Chitin, an extracellular polysaccharide, is synthesized by membrane-embedded chitin synthase (CHS) utilizing intracellular substrates. The mechanism of the translocation of synthesized chitin across the membrane to extracellular locations remains unresolved. We prove that the chitin synthase from *Phytophthora sojae* (*Ps*CHS) is a processive glycosyltransferase, which can rapidly produce and tightly bind with the highly polymerized chitin. We further demonstrate that *Ps*CHS is a bifunctional enzyme, which is necessary and sufficient to translocate the synthesized chitin. *Ps*CHS was purified and then reconstituted into proteoliposomes (PLs). The nascent chitin is generated and protected from chitinase degradation unless detergent solubilizes the PLs, showing that *Ps*CHS translocates the newly produced chitin into the lumen of the PLs. We also attempted to resolve the *Ps*CHS structure of the synthesized chitin-bound state, although it was not successful; the obtained high-resolution structure of the UDP/Mn^2+^-bound state could still assist in describing the characterization of the *Ps*CHS’s transmembrane channel. Consistently, we demonstrate that *Ps*CHS is indispensable and capable of translocating chitin in a process that is tightly coupled to chitin synthesis.

## 1. Introduction

Chitin is the second most abundant extracellular polysaccharide on Earth, following cellulose. It is a linear polysaccharide, which is composed of N-acetylglucosamine (GlcNAc) connected by β-(1,4) glycosidic bonds. It serves as a key component of fungal cell walls and insect exoskeletons, providing structural stability and mechanical defense, which are crucial for survival [1,2].

Chitin is synthesized by chitin synthase (CHS), a membrane-integrated protein belonging to the glycosyltransferase family 2, consisting of a glycosyltransferase (GT) domain and a transmembrane (TM) region [3,4,5]. A short N-terminal domain (NTD) exists in some CHSs, like *Phytophthora sojae* chitin synthase 1 (*Ps*CHS1), to stabilize the dimerization. CHS incorporates UDP-N-acetylglucosamine (UDP-GlcNAc) onto the acceptor molecule to form the nascent chitin, which has been validated by in vitro activity assays. Recent structural studies have provided near-atomic-level insights into how the GT domain of CHS recognizes intracellular UDP-GlcNAc and catalyzes chitin synthesis on the cytosolic side of the plasma membrane [6,7,8]. As an extracellular polysaccharide, the synthesized chitin will be secreted to the extracellular destination. Although cellulose synthase and hyaluronan synthase, which are also extracellular polysaccharide synthases, have been proven to be able to integrate the functions of glycosyltransferase and translocase, there is currently no direct experimental evidence, however, to demonstrate whether CHS is a bifunctional enzyme that could synthesize and translocate the produced chitin.

As a polysaccharide synthase, CHS has been proven to possess key mechanistic characteristics, including an inverting mechanism and non-reduced ending elongation. The inversion mechanism is evident since the β-(1,4) glycosidic bonds of chitin are formed by donor sugar, which is UDP-GlcNAc in the α conformation [6,7,8,9]. The *Ps*CHS-(GlcNAc)_3_ complex structure supports the elongation of the chitin from the non-reducing end, as the non-reducing end of the (GlcNAc)_3_ is positioned closer to the reaction chamber. The synthesis mechanism, which has not been characterized for CHS so far, is also an important feature for polysaccharide synthase, which has been described as either a processive or distributive mechanism representing opposite extremes [10,11,12,13,14]. Processive enzymes exhibit a strong affinity for the product, allowing them to remain attached and catalyze successive reactions without dissociating during multiple catalysis rounds [15]. As a result, gradual depletion of the substrate and rapid lengthening of the product will be observed, while intermediate products accumulate minimally [13,14,16,17]. In contrast, distributive synthases dissociate from the product after each catalytic reaction, since there is a higher chance of binding excessive substrate compared with intermediate molecules, resulting in a rapid disappearance of the substrate and the formation of multiple early intermediates [13,18].

*Phytophthora sojae* is a pathogen that causes root and stem rot in soybeans, resulting in estimated global economic losses of USD 1–2 billion annually [19]. *P. sojae* possesses two CHS genes, with *CHS2* being very weakly transcribed across all life cycle stages. Chitin is present only during specific developmental stages, such as in mature sporangia and released zoospores. Disruption of the chitin synthase gene *CHS1* retards vegetative growth and asexual reproduction, significantly reducing the pathogenicity of *P. sojae* [20]. *Ps*CHS1 serves as a promising antifungal target and a valuable model system for CHS research. Therefore, we selected *Ps*CHS1 as the research focus, and it will be referred to as *Ps*CHS throughout the following text.

In this study, we demonstrated that *Phytophthora sojae* chitin synthase (*Ps*CHS) exhibits processivity and functions as a bifunctional enzyme, capable of catalyzing both the synthesis and membrane translocation of chitin. In order to understand the molecular mechanism of chitin translocation, we strived to resolve the structure of *Ps*CHS in complex with chitin. Unfortunately, only the *Ps*CHS structure with the UDP/Mn^2+^ bound was obtained, which represents the competitive substrate binding or post-translocation state. The potential chitin translocation channel was characterized according to the UDP/Mn^2+^ occupied structure with a resolution of 2.94 Å.

## 2. Results

### 2.1. Processivity of PsCHS

**Figure 1:**
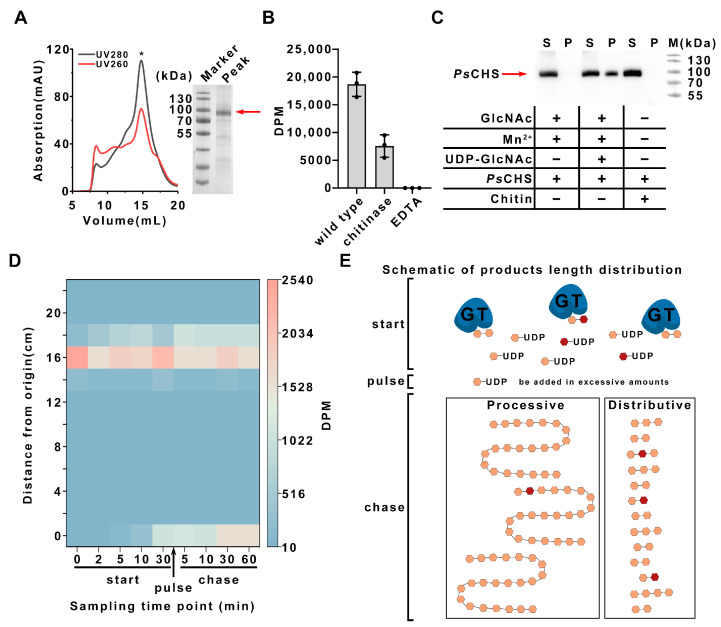
Processivity analysis of *Ps*CHS. (**A**) The size exclusion chromatography profile and SDS–PAGE analysis of purified *Ps*CHS is displayed, with peak position indicated by the asterisk and purified *Ps*CHS marked by the red arrow. (**B**) The purified *Ps*CHS shows activity in vitro (Column 1). Chitinase was added after the synthesis reaction, proving that the synthesized product is chitin (Column 2). Incubation with EDTA can inactivate *Ps*CHS (Column 3). (**C**) The processive property of *Ps*CHS was verified using a sedimentation experiment assay. Western blot analysis indicates that *Ps*CHS is present in the precipitate (P) after the chitin synthesis reaction (Lane 4). *Ps*CHS does not self-precipitate (Lane 2) or interact with the exogenous chitin (Lane 6) and only exists in the supernatant (S) in the control experiments (Lanes 1 and 5). S and P refer to the supernatant and precipitate of centrifugation, respectively. (**D**) The pulse-chase analysis of *Ps*CHS. The pulse samples were extracted from a reaction mixture with a low enzyme-to-substrate molar ratio of 1:3 at a specific time. Then, the enzyme-to-substrate molar ratio was increased to 1:1000 by supplementing the reaction mixture with additional substrate and continuing incubation. The chase samples were removed at the indicated time. The distribution of products was analyzed by scintillation counting after filter paper chromatography. DPM, disintegrations per minute. (**E**) Scheme illustrating the distribution of product lengths for processive and distributive glycosyltransferases. The monosaccharide units are depicted as hexagons, with labeled sugars in brown and unlabeled sugars in orange.

The purified *Ps*CHS (Figure 1A) exhibits chitin synthesis activity in vitro (Figure 1B, column 1), and the product is degraded by chitinase (Figure 1B, column 2). To investigate the processivity of CHS, the purified *Ps*CHS in detergent micelles was subjected to sedimentation experiment and pulse-chase experiment. For the sedimentation experiment, *Ps*CHS was incubated with GlcNAc/Mn^2+^/UDP-GlcNAc at 30 °C for 30 min. The reaction system became turbid due to the production of highly polymerized chitin by *Ps*CHS, which could be further precipitated upon centrifugation because of chitin’s low solubility (Figure S1). Western blot analysis revealed the presence of *Ps*CHS in the precipitation, indicating that *Ps*CHS was closely associated with the synthesized chitin. The control experiment clearly ruled out the possibility that the precipitation of *Ps*CHS comes from the instability of *Ps*CHS or nonspecific binding to exogenous chitin (Figure 1C). These results show that the synthesized chitin remains associated with *Ps*CHS during multiple rounds of catalysis., indicating the processive feature of *Ps*CHS.

The pulse-chase experiments were conducted by introducing a new batch of substrates after a period of reaction. Initially, a substrate-to-enzyme concentration ratio of 3:1 was established, with isotopically labeled substrates added for tracking product distribution. After 30 min incubation, a new batch of unlabeled substrates with a molar ratio relative to *Ps*CHS of 1000 was introduced into the reaction system. Product distribution analysis shows that the labeled substrates start to accumulate in products with a high degree of polymerization at the start stage, and unreacted labeled substrates are incorporated into highly polymerized chitin upon the addition of excess substrate. These products remain in the origin region of filter paper chromatography, which indicates that the synthesized chitin is highly polymerized (Figure 1D). The product distribution indicates that highly polymerized products form even when a substantial amount of substrate remains, with no intermediate-length products detected (Figure 1D). These findings are consistent with the processive mechanism model of glycosyltransferase (Figure 1E), where the enzyme continues to act on the polymer produced from the first batch of substrates without being interrupted by the addition of new substrates. In contrast, a distributive enzyme dissociates from the product between catalysis rounds, allowing equal access for both the first and second substrate batches and resulting in shorter polymers. Combining the sedimentation experiment results with the pulse-chase experiment results, the processive property of *Ps*CHS is clearly demonstrated, which is also necessary for the bifunctional polysaccharide synthase that could synthesize and translocate the produced polysaccharide across the membrane.

### 2.2. Translocation of Chitin by PsCHS

**Figure 2:**
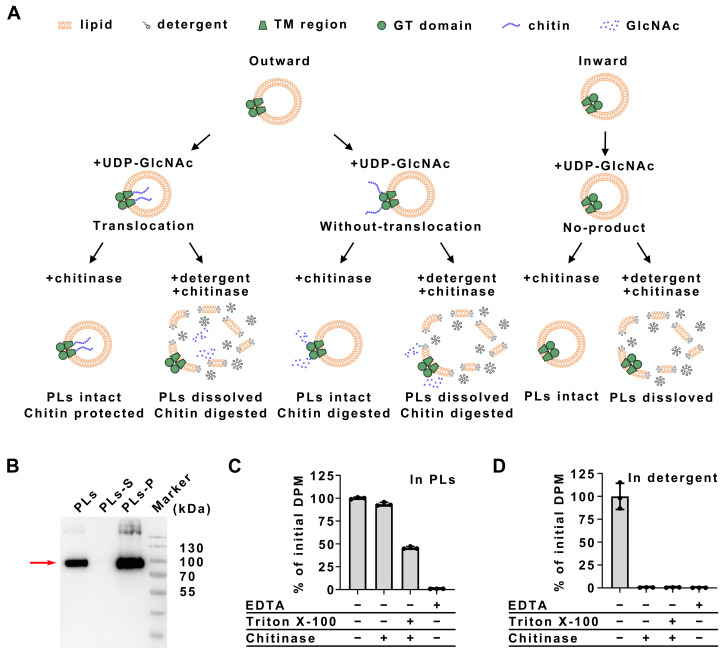
Chitin translocation assay using PsCHS. (**A**) Schematic representation of the in vitro chitin translocation assay. Chitin is only synthesized when the GT domain is outward facing but not inward facing. Translocation of chitin across the vesicle membrane separates the polymer from the solvent, which can be quantified by the enzymatic degradation of chitin. Without translocation, chitin will be susceptible to digestion by chitinase addition, whereas translocated chitin will only be digested after solubilizing the lipid vesicles with detergent. (**B**) Western blot of proteoliposomes (PLs) embedding *Ps*CHS, with positive bands of *Ps*CHS indicated by the red arrow. Western blot analysis indicates that *Ps*CHS co-precipitates with PLs after ultracentrifugation, demonstrating that purified *Ps*CHS was successfully reconstituted into *E. coli* total lipid extract PLs. PLs, *Ps*CHS embedded proteoliposomes; PLs-S, supernatant part after *Ps*CHS embedded proteoliposomes ultracentrifugation; PLs-P, precipitate part after *Ps*CHS embedded proteoliposomes ultracentrifugation. (**C**) In vitro chitin translocation function of *Ps*CHS. PLs containing *Ps*CHS were incubated with a radioactively labeled substrate, and then, the reaction was terminated with EDTA. Subsequently, the PLs were incubated with chitinase in the presence or absence of Triton X-100 followed by inactivation of chitinase with SDS. No chitin is synthesized when EDTA is added to the reaction mixture before *Ps*CHS addition (column 4), confirming that EDTA efficiently terminates chitin elongation. Chitin is quantified relative to the amount obtained without enzymatic digestion. (**D**) Degradation of chitin within the detergent environment. Similar experiments to panel C were conducted, except that *Ps*CHS in detergent was used instead of *Ps*CHS embedded in PLs. Chitin was completely digested in either the presence or absence of Triton X-100.

The proteoliposomes (PLs) synthesis assay were performed to demonstrate the membrane translocation capabilities of *Ps*CHS. Only the GT domain outward confirmation of *Ps*CHS could access the substrate and generate chitin. Furthermore, if *Ps*CHS is able to translocate chitin across the membrane, the synthesized chitin will accumulate in the lumen of PLs and will not be digested by chitinase unless the vesicles are solubilized with detergent. In contrast, if *Ps*CHS was only capable of synthesizing chitin without translocating it across the membrane, the chitin would be located on the outer side of the PLs and thus accessible to chitinase digestion (Figure 2A). The purified *Ps*CHS was successfully reconstituted into PLs (Figure 2B), where it synthesized chitin while mixed with the substrate UDP-GlcNAc (Figure 2C, column 1). After terminating the chitin elongation reaction with EDTA, 20 μU chitinase from *Streptomyces griseus* was added, either in the presence or absence of 2% Triton X-100. As shown in column 2 of Figure 2C, over 90% of the chitin remained undigested when *Ps*CHS embedded in PLs was exposed to chitinase. On the contrary, approximately 60% of chitin was digested when both chitinase and Triton X-100 were present simultaneously (Figure 2C, column 3). As a control, chitin synthesized by *Ps*CHS in a detergent environment was completely digested by chitinase, regardless of the presence of Triton X-100, as the chitin was fully accessible to the chitinase (Figure 2D). Consequently, the observation that chitin produced by *Ps*CHS embedded in PLs is shielded from chitinase digestion indicates that *Ps*CHS could translocate the synthesized chitin into the lumen of the PLs.

### 2.3. Coupling of Chitin Synthesis and Membrane Translocation

**Figure 3:**
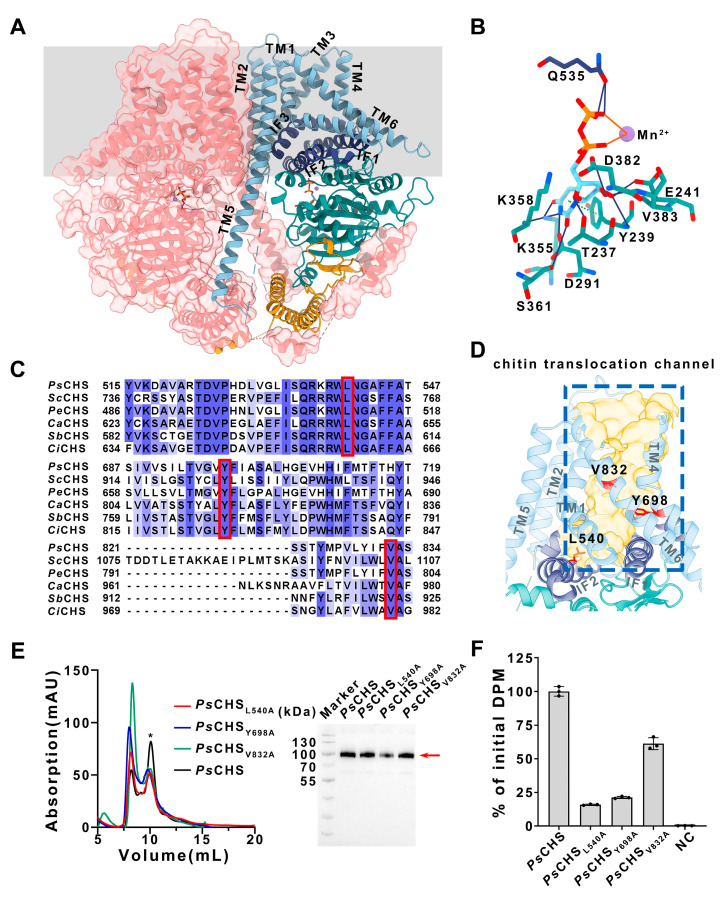
The structure and chitin translocation channel of *Ps*CHS. (**A**) The architecture of the *Ps*CHS. The side view of the *Ps*CHS dimer is shown in surface and cartoon representations. The TM helices, IF helices, GT domain, and N-terminal domain associated with dimerization are colored as pale blue, indigo blue, dark turquoise, and golden yellow, respectively. The other protomer is colored as pink. The unresolved region is shown as dashed lines. The approximate position of the membrane is marked with grey shading. (**B**) Interactions between *Ps*CHS and UDP/Mn^2+^. UDP is stabilized in the reaction cavity of the GT domain through hydrogen bonds and π-π stacking hydrophobic interaction. Mn^2^⁺ coordinates with the pyrophosphate group of UDP. The interactions between *Ps*CHS and UDP/Mn^2+^ were analyzed using Protein–Ligand Interaction Profiler (PLIP) [21]. Residues are colored according to their respective functional regions, consistent with Figure 3A. Hydrogen bonds, π-π stacking interactions, and coordinates between *Ps*CHS and UDP/Mn^2+^ are indicated with blue lines, green dashed lines, and orange lines, respectively. (**C**) Sequence alignment of *Ps*CHS with chitin synthases from other species shows that three residues potentially involved in chitin translocation in *Ps*CHS are conserved among chitin synthases from *Saccharomyces cerevisiae* (*Sc*), *Peronospora effusa* (*Pe*), *Candida albicans* (*Ca*), *Sporothrix brasiliensis* (*Sb*), and *Coccidioides immitis* (*Ci*). These conserved residues are marked with red squares and highlighted in panel D. (**D**) The chitin translocation channel of *Ps*CHS. The presumed chitin translocation channel is illustrated with a golden surface, and the selected conserved amino acids at different positions are shown by sticks. The colors match those in panel A, with the selected residues highlighted in red. (**E**) Purification of *Ps*CHS mutations. The size exclusion chromatography profile for *Ps*CHS and its mutants was obtained using a Superdex 200 Increase 10/300 GL column. The lines in red, blue, green, and black represent the mutants L540A, Y698A, V832A, and wild-type *Ps*CHS, respectively, with peak position indicated by the asterisk. The positive bands in Western blot of purified *Ps*CHS and its mutants is indicated by the red arrow. (**F**) The effect of mutations on *Ps*CHS. Mutating the selected amino acids significantly reduced the activity of chitin synthase.

The structure of the product-bound glycosyltransferase is essential for investigating the mechanism of polysaccharide translocation across membranes. To capture the structure of chitin-bound *Ps*CHS, we incubated the enzyme in the presence of UDP-GlcNAc and Mn^2+^ for 15 min or 40 min before cryo-EM sample preparation. However, only a UDP- and Mn^2+^-occupied structure was resolved for both samples with overall resolutions of 3.20 Å and 2.94 Å, respectively (Figure S2). *Ps*CHS forms a dimer, as previously reported [2]. Each protomer contains a long transmembrane helix, with its N-terminal region extending to the adjacent protomer, stabilizing the dimer with the interaction interface located at the GT domain as well as the TM region (Figure 3A). UDP and Mn^2+^ are positioned in the reaction cavity of the GT domain, where *Ps*CHS stabilizes UDP via hydrogen bonding and π-π stacking interactions. Q535 of the amphipathic interface helices (IF2) forms two hydrogen bonds with the pyrophosphate group, while other hydrogen bonds occur between the uridine portion of UDP and the GT domain. Specifically, E241, D382, V383, T237, and Y239 form hydrogen bonds with the ribose moiety, while D291, K355, K358, and S361 bond with the uracil moiety. Y239 also participates in π-π stacking with the uracil ring and Mn^2^⁺ coordinates with the pyrophosphate group (Figure 3B). The GT domain of the *Ps*CHS protomer adopts a GT-A fold and packs against the TM region, which consists of six transmembrane helices (TM1-6) and three amphipathic interface helices (IF1-3). IF1 and IF2 connect the GT domain and transmembrane helices, framing the entrance of the potential chitin translocation channel. IF3 adopts an arched configuration over the IF1-IF2 pair and collectively creates the potential transmembrane channel with TM1, TM3, TM4, and TM6 (Figure 3A). The residues surrounding and pointing to the potential transmembrane channel are likely to interact with the synthesized chitin chains.

In membrane-integrated glycosyltransferases, the synthesis and translocation of polysaccharides across the membrane are thought to be closely related processes. Therefore, blocking the translocation of the polysaccharide product could directly inhibit the activity of the polysaccharide synthase. To prove this hypothesis, sequence alignment of chitin synthases of different species identified highly conserved residues involved in forming the transmembrane channels. The high conservation of these residues suggests that they may play a critical role in the translocation function of chitin synthases. Three conserved residues, L540 of IF2, Y698 of TM4, and V832 of TM6, were selected for mutation to assess their impact on *Ps*CHS activity (Figure 3C). These selected residues are located at different positions within the transmembrane channel with L540 at the entrance, Y698 at one-third of the distance from the entrance, and V832 at the middle position of the channel, respectively (Figure 3D). The purified variants (Figure 3E) were subjected to in vitro enzyme activity assays and exhibited a significant decrease compared with the wild type (Figure 3F). As shown in column 4 of Figure 3D, mutating V832 to tyrosine resulted in an approximately 40% reduction in chitin synthase activity. And over 75–80% of the chitin synthase activity was lost when L540 or Y698 were mutated to alanine (Figure 3F, columns 2 and 3). These results suggest that these conserved residues interact with the synthesized chitin polymer and are involved in chitin translocation. Mutation of these residues will cause the loss of chitin synthesis activity, indicating that *Ps*CHS couples chitin synthesis with its membrane translocation.

## 3. Discussion

Processive mechanisms have been identified in numerous biological processes, including nucleic acid replication, transcription, and translation [22]. The tight association between the enzyme and its products is a hallmark characteristic of a processive enzyme, as the retained products near the active site allow the enzyme to easily perform subsequent rounds of catalysis. Several extracellular polysaccharide synthases, such as cellulose synthase and hyaluronan synthase, have been proven to be processive enzymes [23,24,25]. In the study presented herein, the sedimentation experiment and pulse-chase experiment were conducted to clearly show that *Ps*CHS is also a processive enzyme. The processivity of glycosyltransferases is thought to be closely related to polysaccharide translocation activity, allowing for the export of the growing polysaccharide chain. Although chitin synthase, the same as cellulose synthase and hyaluronan synthase, is a processive membrane-integrated glycosyltransferase, the chitin translocation properties of chitin synthase cannot be definitively confirmed until direct experimental results are demonstrated. This is because peptidoglycan glycosyltransferase, while also being a membrane-integrated glycosyltransferase, is known to be a processive enzyme despite lacking membrane translocation activity [14]. Therefore, the membrane translocation assay with *Ps*CHS embedded in proteoliposomes described herein has demonstrated that it functions as a bifunctional enzyme, capable of both synthesizing and translocating chitin.

The structure of *Ps*CHS shows a potential chitin translocation channel, which exerts a comparable size with cellulose synthase and hyaluronan synthase. According to research on cellulose synthase and hyaluronan synthase, polysaccharides in the transmembrane channel interact with the residues generally through π-π stacking interaction, π-CH stacking interaction, and hydrogen bonds [24,25,26,27]. Therefore, some aromatic residues with side chains oriented toward the transmembrane channel, such as Y698, may interact with the synthesized chitin chain via π-π stacking interactions or hydrogen bonds. Meanwhile, the non-polar side chains of residues such as L540 and V832 may interact with the chitin chain through π-CH stacking or hydrophobic interaction, as their mutations negatively affect chitin synthase activity.

Polysaccharide synthase could recognize the specific nucleoside diphosphate-activated sugar substrate. Substrate recognition not only occurs in the GT domain but also in the TM region of polysaccharide synthase. Mutations in residues within the transmembrane channel will weaken the interactions with the sugar chains and could further reduce chitin synthesis activity. As shown from the sequence alignment of chitin synthase, cellulose synthase, and hyaluronan synthase, although generally, the residues of the TM region are more variable, certain residues are conserved in chitin synthases but not in other polysaccharide synthases. In *Ps*CHS, tyrosine at positions 698 and 718 are conserved in chitin synthase, while these positions are occupied by leucine in cellulose synthase and methionine in hyaluronan synthase (Figure S3). Based on the structural difference between the sugar units of chitin (GlcNAc), cellulose (Glc), and hyaluronan (GlcA and GlcNAc), these amino acids may play a role in interactions with acetamido groups, which is the unique group in chitin (GlcNAc) among these three sugars. Although the mutation experiments described herein prove that residues 698 and 718 [7] are important for activity, the structure of *Ps*CHS with synthesized chitin in the transmembrane channel is required to confirm these interactions.

## 4. Materials and Methods

### 4.1. Protein Expression and Purification

*Ps*CHS and its variants were expressed and purified as previously described [6], with minor modifications. HEK293F cells were transfected with a pcDNA3.1 vector containing the *Ps*CHS gene fused with a C-terminal 6 × histidine tag and cultured for 72 h to express *Ps*CHS. Following culture harvesting, cells were resuspended in resuspension buffer (25 mM Tris-HCl pH 8.0 and 150 mM NaCl) and lysed using a chilled high-pressure homogenizer (Union-Biotech, Shanghai, China). Subsequently, the lysate was centrifuged at 12,500 rpm for 20 min at 4 °C in an R20A2 rotor (Hitachi, Tokyo, Japan) to remove unbroken cells and aggregated materials. Membranes were collected by ultracentrifugation of the supernatant at 200,000× *g* in a Ti45 rotor (Beckman Coulter, Brea, CA, USA) for 1 h at 4 °C, followed by solubilization in resuspension buffer supplemented with 1% digitonin (Biosynth, Compton, UK) overnight. After ultracentrifugation at 200,000× *g* for 30 min at 4 °C, the supernatant was incubated with Ni-NTA resin for 1 h at 4 °C. The column was sequentially washed with wash Ⅰ buffer (25 mM Tris-HCl pH 8.0, 1 M NaCl, and 20 mM imidazole) and wash Ⅱ buffer (resuspension buffer supplemented with 40 mM imidazole). Elution of *Ps*CHS was performed using resuspension buffer supplemented with 250 mM imidazole. The eluted fraction was concentrated and injected onto a Superose 6 Increase 10/300 GL column (Cytiva, Wilmington, DE, USA) equilibrated with resuspension buffer for further purification. All purification buffers above contained 0.05% digitonin. Peak fractions were collected, concentrated, and subsequently analyzed using SDS-PAGE and Western blot.

### 4.2. Activity Assays

To investigate the activity of *Ps*CHS, 5 μL 1 mg/mL purified *Ps*CHS was added to 15 μL reaction buffer containing 25 mM Tris pH 8.0, 150 mM NaCl, 10 mM MnCl_2_, 2 mM GlcNAc, and 2 mM UDP-GlcNAc and supplemented with 0.5 μCi ^3^H-labelled UDP-GlcNAc (PerkinElmer, Shelton, CT, USA) for product tracing. Following 30 min incubation at 30 °C, 40 mM EDTA was added to the reaction system to terminate the reaction. To detect the synthesis products, the reaction mixture was subjected to digestion with 10 μU chitinase from *Streptomyces griseus* (Sigma-Aldrich, Burlington, MA, USA) at 30 °C for 10 min. Subsequently, 2% SDS was added to denature the chitinase. *Ps*CHS was incubated with 40 mM EDTA and 2% SDS as a negative control. It was then spotted onto Whatman 3MM filter paper and developed using a solvent consisting of 65% 1 M ammonium acetate (pH5.5) and 35% ethanol. Under these conditions, highly polymerized chitin remained at the origin position; the dried origins were cut into 2 cm strips, and radioactivity was counted in Ultima Gold scintillation fluid using a liquid scintillation counter (PerkinElmer, Shelton, CT, USA).

### 4.3. Processivity Analysis

For the sedimentation experiment, the reaction system was scaled up four-fold. After 30 min incubation at 30 °C, the reaction mixture was centrifuged at 15,000× *g* for 10 min at 4 °C. Both the supernatant and the precipitate were analyzed using Western blot. The reaction mixture which omits UDP-GlcNAc and the mixture where *Ps*CHS is incubated with exogenous chitin from shrimp were both involved as negative controls.

For pulse-chase analysis [28], 0.5 nmol purified *Ps*CHS was incubated with 50 nmol GlcNAc, 50 nmol Mn^2+^, 1.5 nmol UDP-GlcNAc, and 0.0019 nmol ^3^H-labelled UDP-GlcNAc at 30 °C in 200 μL reaction mixture. Then, a 10 μL sample was collected after 2, 5, 10, and 30 min, respectively, followed by adding 375 nmol UDP-GlcNAc to continue incubation. Another 10 μL sample was collected after 5, 10, 30, and 60 min during the chase. At each time point, 2% SDS/40 mM EDTA was added to the sample to terminate the reaction and release products. Then, the paper chromatogram and liquid scintillation counting were performed as in the activity assays.

### 4.4. Proteoliposome (PL) Reconstitution

The *E. coli* total lipid extract (Avanti research, Alabaster, AL, USA) at a quantity of 12.5 mg was dissolved in 1 mL chloroform, and the chloroform was evaporated by nitrogen purging. After lyophilization overnight, 1 mL of 60 mM DDM (Anatrace, Maumee, OH, USA) was added with vibration to dissolve the lipid. The lipid and purified *Ps*CHS were mixed at a quality ratio of 4:1 and incubated at 4 °C for 30 min. Bio-Beads SM-2 Resin (Bio-Rad, Hercules, CA, USA) was then added and replaced every hour to remove the detergent until the mixture became turbid, indicating the completion of lipid vesicle assembly.

### 4.5. Chitin Translocation in PLs

The translocation experiment of chitin refers to the hyaluronan translocation experiment with some modifications [29]. First, 5 μL of PLs was added to 15 μL of reaction buffer, as described for the activity assays. After incubation at 30 °C for 5 min, the synthesis reaction was terminated by adding 40 mM EDTA. Following that, the samples were subjected to digestion with 20 μU chitinase at 30 °C for 10 min, either with or without 2% Triton X-100. Subsequently, 2% SDS was added to denature the chitinase and dissolve the PLs. Then, radioactivity was quantified as described above. As a control, PLs were replaced by purified *Ps*CHS to eliminate the possibility that Triton X-100 merely increases the susceptibility of chitin to chitinase digestion.

### 4.6. Cryo-EM Sample Preparation and Data Collection

*Ps*CHS was concentrated to 3 mg/mL and incubated with 0.5 mM UDP-GlcNAc and 1 mM Mn^2+^ for 15 min or 40 min at 25 °C. Then, 10 μL samples were loaded onto glow-discharged grids with holey carbon film (Quantifoil, Au 300 mesh, R1.2/1.3). The grids were blotted for 2 s at 4 °C and 100% humidity and were then flash-frozen in liquid ethane using Vitrobot (Thermo Fisher Scientific, Waltham, MA, USA).

Data collection for *Ps*CHS samples incubated for 15 min (*Ps*CHS15) and 40 min (*Ps*CHS40) was conducted utilizing a Titan Krios 300 kV electron microscope (Thermo Fisher Scientific, Waltham, MA, USA) equipped with a K3 direct detection camera (Gatan, Pleasanton, CA, USA) for *Ps*CHS15 and Felcon 4i direct detection camera (Thermo Fisher Scientific, Waltham, MA, USA) for *Ps*CHS40 at Shandong University. Images of *Ps*CHS15 were acquired using an EPU (Thermo Fisher Scientific, Waltham, MA, USA) at a nominal magnification of ×81,000 and recorded in super-resolution mode with a pixel size of 1.04 Å. The total dose of 60 e^−^/Å^2^ was distributed over 32 frames, and the target defocus ranged from −0.8 to −1.8 μm. Images of *Ps*CHS40 were captured under similar conditions, with a nominal magnification of ×130,000 and a pixel size of 0.92 Å. The collection utilized counting mode, with a total dose of 60 e^−^/Å^2^ spread across 31 frames and a target defocus ranging from −1.0 to −2.0 μm.

### 4.7. Data Processing

CryoSPARC was selected for data processing. Movies were imported, followed by motion correction and contrast transfer function estimation. To generate references for template-based particle picking, particles were selected from the initial round of 2D classification based on blob-picking particles. Subsequently, particles extracted from template-based picking underwent another round of 2D classification followed by ab-initio reconstruction and heterogeneous refinement. High-resolution reconstructions were produced using homogeneous refinement and non-uniform refinement. The overall resolution of the final *Ps*CHS map was determined using gold-standard Fourier shell correlation. For the *Ps*CHS15 and *Ps*CHS40 datasets, the final reconstructions yielded an average resolution of 3.20 Å and 2.94 Å, respectively, from 255,525 and 561,882 particles.

### 4.8. Model Building

The two structures were built by similar procedures. The apo-state structure of *Ps*CHS1 [6] was chosen as the initial model and corrected in COOT [30]. Structural refinement was carried out using the phenix.real_space_refine application within PHENIX [31]. The final model of *Ps*CHS with 15 min incubation lacks residues 1–22 and 40–90 in the N-terminus and residues 738–761 and 860–925 in the C-terminus. The final model of *Ps*CHS with 40 min incubation lacks residues 1–22, 40–48, 52–76, and 88–90 in the N-terminus and residues 738–761 and 860–925 in the C-terminus. All of the figures were prepared in ChimeraX [32].

## 5. Conclusions

The extracellular polysaccharide chitin is synthesized by chitin synthase (CHS), a membrane-integrated protein thought to transport chitin across the membrane to the extracellular space. However, direct experimental evidence has remained limited. In this study, through pulse-chase and sedimentation experiments, we demonstrated that chitin synthase can synthesize chitin in a processive manner. Using reconstituted proteoliposomes (PLs), we further confirmed the transmembrane transport function of chitin synthase. As expected, mutating conserved residues in the transmembrane channel verified the coupling of chitin synthesis and translocation functions. Notably, chitin synthase, along with cellulose synthase and hyaluronan synthase, belongs to the family of extracellular polysaccharide synthases. This work may contribute to a broader understanding of other extracellular polysaccharide synthases. Furthermore, detailed biochemical and biophysical analyses may be required to explore the mechanisms of the coupling between chitin synthesis and membrane translocation processes.

## Data Availability

The atomic model and EM map for the structures of *Ps*CHS with 15 min and 40 min incubation have been deposited in the RCSB PDB and the EMDB database with accession codes 8Z0O and EMD-39709 and 8K52 and EMD-36893, respectively.

## Supplementary Materials

The following supporting information can be downloaded at: https://www.mdpi.com/article/10.3390/ijms252111667/s1.
