# Supplementary material for: Chitin Translocation Is Functionally Coupled with Synthesis in Chitin Synthase
Source: Int J Mol Sci. 2024 Oct 30;25(21):11667. doi: 10.3390/ijms252111667 (PMC11546553; doi:10.3390/ijms252111667)
Supplement: Supplementary file 1 [file ijms-25-11667-s001.zip › ijms-3272099-supplementary.pdf]

Supplementary figures:

Figure S1

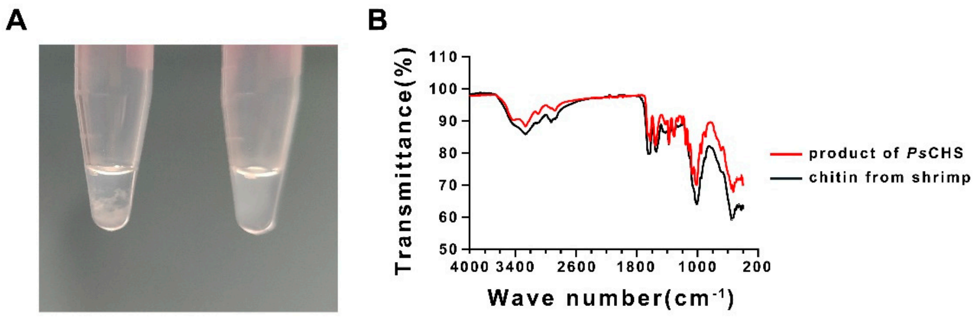

Figure S1 Product of recombinant *Ps*CHS. **(A)**. *In vitro* activity assay of recombinant *Ps*CHS. The left tube shows precipitate formation in the presence of the substrate UDP-GlcNAc, while the right tube, which lacks UDP-GlcNAc, remains clear and serves as a control. **(B)**. Fourier Transform Infrared (FTIR) spectra comparing chitin from shrimp (black line) with product of *Ps*CHS (red line). The spectra illustrate the characteristic absorption bands of chitin in both samples.

Figure S2

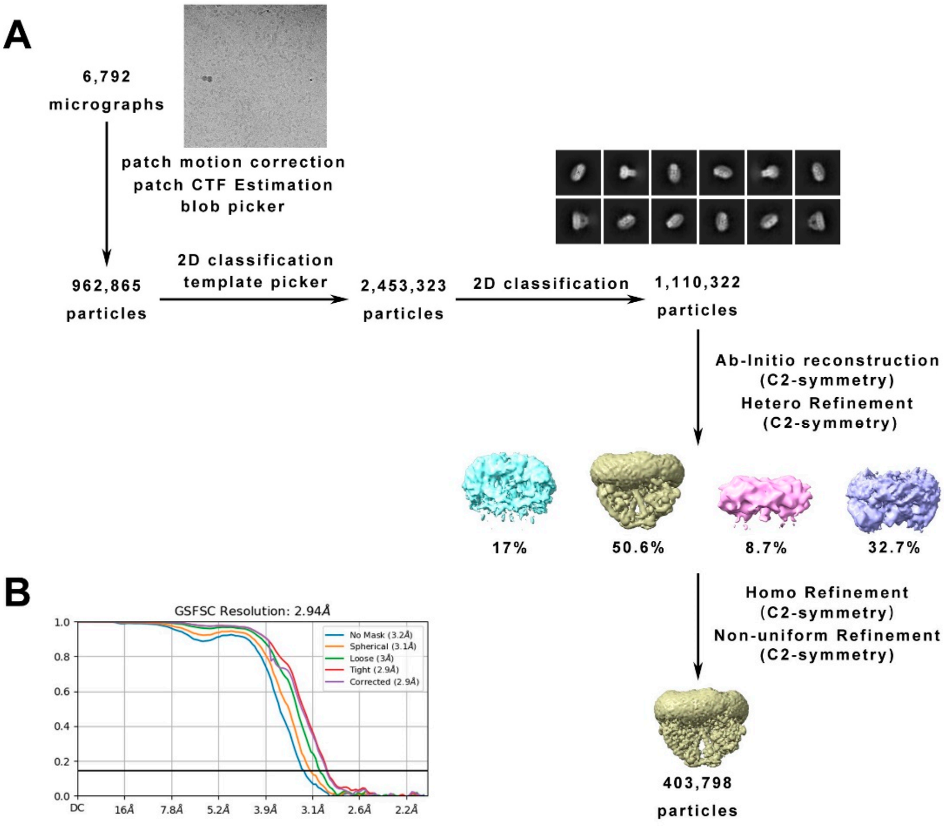

Figure S2 Cryo-EM data process of *Ps*CHS-UDP/Mn^{2+} complex. **(A)** Flowchart of data processing for *Ps*CHS-UDP/Mn^{2+} complex. **(B)** Gold-standard Fourier shell correlation (GSFSC) curves of *Ps*CHS-UDP/Mn^{2+} complex generated by Phenix. The date processing for *Ps*CHS with 15 mins and 40 mins incubation was similar and only the 40 mins results are shown.

Figure S3

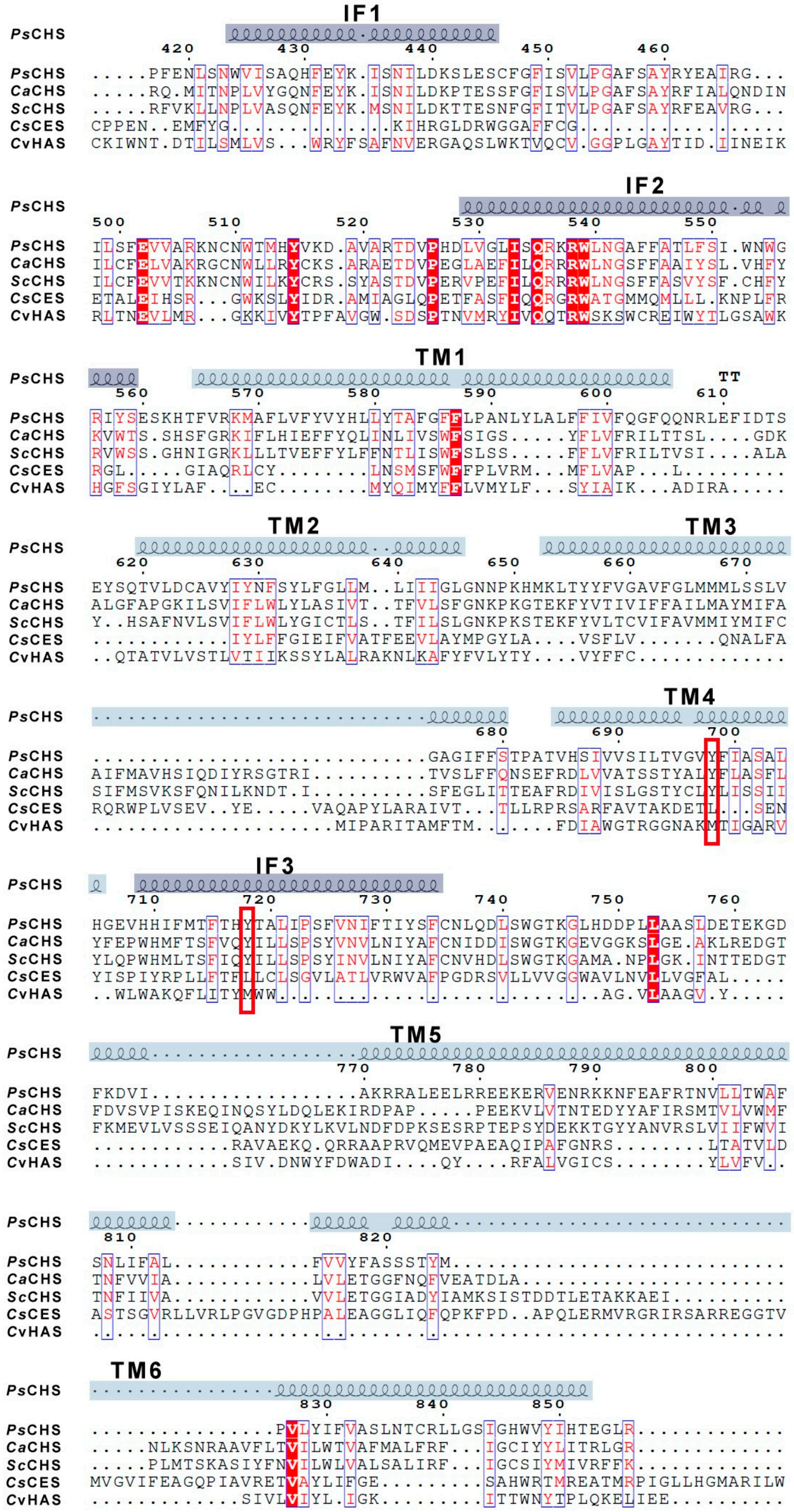

IF1
PsCHS
420 430 440 450 460
PsCHS .....PFENLSNWVISAQHFEYK.ISNILDKSLESCFGFISVLPGAFSAYRYEAIRG...
CaCHS .....RQ.MITNPLVYGQNFEYK.ISNILDKPTESSFGFISVLPGAFSAYRFIALQNDIN
ScCHS .....RFVKLLNPLVASQNFEYK.MSNILDKTTESNFGFITVLPGAFSAYRFEAVRG...
CsCES CPPEN..EMFYG.............KIHRGLDRWGGAFFCG....................
CvHAS CKIWNT.DTILSMLVS...WRYFSAFNVERGAQSLWKTVQCV.GGPLGAYTID.IINEIK
IF2
PsCHS
500 510 520 530 540 550
PsCHS ILSFEVVARKNCNWTMHYVKD.AVARTDVPHDLVGLISQRKRWLNGAFFATLFSI.WNWG
CaCHS ILCFELVAKRGCNWLLRYCKS.ARAETDVPEGLAEFILQRRRWLNGSFFAAIYSL.VHFY
ScCHS ILCFEVVTKKNCNWILKYCRS.SYASTDVPERVPEFILQRRRWLNGSFFASVYSF.CHFY
CsCES ETALEIHSR...GWKSLYIDR.AMIAGLQPETFASFIQQRGRWATGMMQMLLL.KNPLFR
CvHAS RLTNEVLMR...GKKIVYTPFAVGW.SDSPTNVMRYIVQQTRWSKSWCREIWYTLGSAWK
TM1
PsCHS
TT
560 570 580 590 600 610
PsCHS RIYSESKHTFVRKMAFLVFYVYHLLYTAFGFFLPANLYLALFFIVFQGFQQNRLEFIDTS
CaCHS KVWTS.SHSFGRKIFLHIEFFYQLINLIVSWFSIGS.....YFLVFRILTTSL....GDK
ScCHS RVWSS.GHNIGRKLLLTVEFFYLFFNTLISWFSLSS.....FFLVFRILTVSI....ALA
CsCES RGL.....GIAQRLCY........LNSMSFWFFPLVRM...MFLVAP...L.........
CvHAS HGFSGIYLAF...EC........MYQIMYFFLVMYLF...SYIAIK...ADIRA.....
TM2
TM3
PsCHS
620 630 640 650 660 670
PsCHS EYSQTVLDCAVYIYNFSYLFGLLM..LIIIGLGNNPKHMKLTYYFVGAVFGLMMMLSSLV
CaCHS ALGFAPGKILSVIFLWLYLASIVT..TFVLSFGNKPKGTEKFYVTIVIFFAILMAYMIFA
ScCHS Y..HSAFNVLSVIFLWLYGICTLS..TFILSLGNKPKSTEKFYVLTCVIFAVMMIYMIFC
CsCES ............IYLFFGIEIFVATFEEVLAYMPGYLA....VSFLV.......QNALFA
CvHAS ..QTATVLVSTLVTIIKSSYLALRAKNLKAFYFVLYTY....VYFFC............
TM4
PsCHS
680 690 700
PsCHS ............................GAGIFFSTPATVHSIVVSILTVGVYFIASAL
CaCHS AIFMAVHSIQDIYRSGTRI.........TVSLFFQNSEFRDLVVATSSTYALYFLASFL
ScCHS SIFMSVKSFQNILKNDT.I..........SFEGLITTEAFRDIVISLGSTYCLYLISSII
CsCES RQRWPLVSEV..YE.....VAQAPYLARAIVT...TLLRPRSARFAVTAKDETL...SEN
CvHAS ....................MIPARITAMFTM..........FDIAWGTRGGNAKMTIGARV
IF3
PsCHS
710 720 730 740 750 760
PsCHS HGEVHHIFMTFTHYTALIPSFVNIFTIYSFCNLQDLSWGTKGLHDDPLLAASLDETEKGD
CaCHS YFEPWHMFTSFVQYILLSPSYVNVLNIYAFCNIDDISWGTKGEVGGKSLGE.AKLREDGT
ScCHS YLQPWHMLTSFIQYILLSPSYINVLNIYAFCNVHDLSWGTKGAMA.NPLGK.INTTEDGT
CsCES YISPIYRPLLFTFLLCLSGVLATLVRWVAFPGDRSVLLVVGGWAVLNVLLVGFAL.....
CvHAS ..WLWAKQFLITYMWW..........................AG.VLAAGV.Y.....
TM5
PsCHS
770 780 790 800
PsCHS FKDVI.................AKRRALEELRREEKERVENRKKNFEAFRTNVLLTWAF
CaCHS FDVSVPISKEQINQSYLDQLEKIRDPAP.....PEEKVLVTNTEDYYAFIRSMTVLVWMF
ScCHS FKMEVLVSSSEIQANYDKYLKVLNDFDPKSESRPTEPSYDEKKTGYYANVRSLVIIFWVI
CsCES ............RAVAEKQ.QRRAAPRVQMEVPAEAQIPAFGNRS........LTATVLD
CvHAS ............SIV.DNWYFDWADI....QY....RFALVGICS........YLVFV..
PsCHS
810 820
PsCHS SNLIFAL............FVVYFASSSTYM............................
CaCHS TNFVVIA............LVLETGGFNQFVEATDLA......................
ScCHS TNFIIVA............VVLETGGIADYIAMKSISTDDTLETAKKAEI.........
CsCES ASTSGVRLLVRLPGVGDPHPALEAGGLIQFQPKFPD..APQLERMVRGRIRSARREGGTV
CvHAS ....
TM6
PsCHS
830 840 850
PsCHS ................PVLYIFVASLNTCRLLGSIGHWVYIHTEGLR............
CaCHS .....NLKSNRAAVFLTVILWTVAFMALFRF...IGCIYYLITRLGR............
ScCHS .....PLMTSKASIYFNVILWLVALSALIRF...IGCSIYMIVRFFK............
CsCES MVGVIFEAGQPIAVRETVAYLIFGE.........SAHWRTMREATMRPIGLLHGMARILW
CvHAS ..............SIVLVIYL.IGK.........ITTWNYTPLQKELIEE.........

Figure S3 Sequence alignment of the transmembrane channel of polysaccharide synthases. Sequences of *Phytophthora sojae* chitin synthase (*Ps*CHS), *Candida albicans* chitin synthase (*Ca*CHS), *Saccharomyces cerevisiae* chitin synthase (*Sc*CHS), *Cereibacter sphaeroides* cellulose synthase (*Cs*CES) and *Chlorella virus* hyaluronan synthase (*Cv*HAS) was obtained from the RCSB PDB with accession codes 7WJM, 7STL, 8K3Q, 4HG6 and 7SP7, respectively.
